# Bibliography

**Published:** 1918-09

**Authors:** 


### BIBLIOGRAPHY

### A CHILD'S BOOK OF THE TEETH

This is a small primer about the things a child may understand about his teeth. It is written to convey to the child mind the essential things about the teeth. The story could also be read and studied to advantage by children of older growth. It is fully illustrated with pictures that will aid materially to catch the child's attention and fix in his memory the precepts that should be kept in mind as to the value of the teeth and the necessity for their care. We have seen much more pretentious books with less knowledge in them and which could not compare with this little book in usefulness. It is very well composed by Dr. H. W. Ferguson and is published by The World Book Co. of Yonkers-on-Hudson, New York. It's a good book to present to the dentist's little patients.

### MODERN DENTISTRY FOR THE LAITY AND INDUSTRIAL DENTISTRY

This is the title to a little book written by Alfred A. Crocker of the S. A. Crocker Co., who sets forth in a very concise and practical form the occasion for systematic supervision of tooth health, especially of people engaged in industrial vocations where they are likely to be more or less careless of this important health function, or where the environment is not conducive to oral sanitation. The book presents to business men the fact that it is of decided economic advantage to install a complete dental office in every business where any considerable number of people are employed. Good tooth health means fewer interruptions of business and promotes larger production, as is shown by the large number of concerns that are installing dental offices in their plants.

